# The Association Between Antipsychotics and Bone Fragility: An Updated Comprehensive Review

**DOI:** 10.3390/diagnostics14232745

**Published:** 2024-12-05

**Authors:** Michele Mercurio, Giovanna Spina, Olimpio Galasso, Giorgio Gasparini, Cristina Segura-Garcia, Pasquale De Fazio, Renato de Filippis

**Affiliations:** 1 Department of Orthopaedic and Trauma Surgery, Magna Graecia University, “Renato Dulbecco” University Hospital, V.le Europa, (Loc. Germaneto), 88100 Catanzaro, Italy; michele.mercurio@unicz.it (M.M.); gasparini@unicz.it (G.G.); 2 Research Center on Musculoskeletal Health, MusculoSkeletal Health@UMG, Magna Graecia University, 88100 Catanzaro, Italy; 3 Department of Medicine, Surgery and Dentistry, University of Salerno, 84081 Baronissi, Italy; ogalasso@unisa.it; 4 Psychiatry Unit, Department of Medical and Surgical Sciences, Magna Graecia University, 88100 Catanzaro, Italy; segura@unicz.it; 5 Psychiatry Unit, Department of Health Sciences, Magna Graecia University, 88100 Catanzaro, Italy; defazio@unicz.it (P.D.F.); rdefilippis@unicz.it (R.d.F.)

**Keywords:** adverse drug reaction, antipsychotic, prolactin, bone mineral density, falls, fracture, hip, vertebrae, schizophrenia, pharmacovigilance

## Abstract

Background: Antipsychotic drugs appear to be related to reduced bone mineral density (BMD). We conducted a narrative review to collect the available literature investigating the relationship between antipsychotic use and bone fragility. Methods: A review of the published literature was conducted and reported through PubMed/Scopus/Cochrane libraries. We included studies using any antipsychotic treatment where the bone metabolism, osteoporosis, and/or risk of fractures has been assessed. Results: After screening 1707 items, we finally included 15 papers. A total of 3245 initial patients were identified, of whom 1357 patients with a mean age of 43.8 years underwent antipsychotic treatment and were analyzed. The mean antipsychotic treatment duration of the treated group was 15.8 ± 13.9 years. Among the included studies, two reported a statistically significant difference in lumbar BMD reduction between the antipsychotic exposed group and the control group. Femoral neck BMD levels had been reported in four of the case–control studies; two reported a statistically significant difference in femoral neck BMD reduction between the antipsychotic exposed group and the control group. Conclusions: Prolonged use of antipsychotic treatment seems to be associated with an increased risk of reduced BMD, and, consequentially, with an augmented risk of bone fragility and fractures. This effect is not limited to vulnerable groups, such as those with significant medical comorbidities, the elderly, and postmenopausal women, but may also apply to anyone using antipsychotics in the long-term. Clinicians’ awareness of antipsychotic prescriptions should optimize their potential while reducing this risk.

## 1. Introduction

Antipsychotic (AP) medications are primarily prescribed for major psychiatric disorders, and their use has been seeing an upward trend globally [1,2]. Indeed, in addition to on-label use in schizophrenia and bipolar disorder, the off-label use of these drugs has also expanded, with 40–75% of prescriptions for adults addressing conditions like resistant major depressive disorders, anxiety disorders, insomnia, eating disorders and agitation [3]. In children, in- and off-label use ranges from 36 to 93%, treating issues such as early onset of severe psychiatric conditions, attention-deficit/hyperactivity disorder (ADHD), severe anxiety disorders, and mood disorders [3,4].

While on the one hand APs’ use is not questioned in light of their significant clinical efficacy and overall adequate tolerability, on the other hand, there are concerns about the long-term safety of continuous APs’ use, as they may cause side effects such as extrapyramidal symptoms, orthostatic hypertension, hyperprolactinemia, sedation, sexual dysfunction, and potentially osteoporosis [5,6,7].

Osteoporosis is a progressive skeletal illness defined by weakened bone strength related to low bone mineral density (BMD), which heightens the risk of fractures [8]. Osteoporosis is defined as a BMD T-score of −2.5 or less and osteopenia as a BMD T-score between −1 and −2.5 according to the diagnostic criteria of the WHO [9]. Dual energy x-ray absorptiometry (DXA) is the method used in clinical practice to measure BMD which correlates with fracture risk. It has emerged as a major public health issue and is recognized as the most prevalent bone disease worldwide [10] affecting elderly people, with the highest incidence among females, especially in Europe, the USA, and Japan [11]. Often referred to as a “silent disease”, osteoporosis is typically only identified after a fracture occurs [12].

The literature recognizes several well-established factors related to reduced BMD, thus increasing the risk of osteoporosis and fractures, including the female sex, an older age, a white race, a low body mass index (BMI), smoking, excessive alcohol consumption, a sedentary lifestyle, a family history of the condition, a history of previous fractures, protracted antidepressant use, oral corticosteroid use, or other high-risk medications (e.g., thyroid hormone replacement, immunosuppressant drugs, warfarin), and severe medical disease (i.e., kidney failure, inflammatory bowel disease, rheumatoid arthritis, liver disease, or an eating disorder) [13,14,15,16]. Research indicates that individuals who have experienced a prior fracture are more susceptible to subsequent fractures, resulting in a diminished quality of life, increased morbidity, higher disability rates, and more frequent hospitalizations [17]. The impact of fractures extends beyond health and wellbeing, placing a significant financial strain on the healthcare system [18]. Therefore, there is a pressing need to raise awareness to promote bone health and mitigate the consequences and costs related to fractures [19,20].

There are studies in the literature evaluating the risk of fractures among individuals taking APs, mainly focusing on patients suffering from schizophrenia or dementia [21], but some research has also examined their effects on BMD in other patient groups, such as those with bipolar disorder [22,23] and autism [24]. Overall, a review by Crews and Howes [25] found that patients on APs, regardless of diagnosis, had lower BMD compared to controls. Additionally, AP use has been linked to an increased risk of falls and osteoporotic fractures [26].

In this regard, there are some data coming from large databases, screened to investigate the potential existing link between AP prescription and a higher risk of hip fractures [27]. However, the findings have been inconsistent [28,29,30,31,32,33,34]. Indeed, some studies have reported an increased risk, including those showing a dose–response relationship [32], while others have examined the differing effects of first- and second-generation APs [34]. Additionally, the impact on prolactin levels has been explored [33], with evidence suggesting that long-term AP treatment in patients suffering from schizophrenia reduces BMD [35], raising the risk of osteoporosis [36,37]. One study indicated a heightened fracture risk in the weeks preceding AP drug initiation, implying that patient characteristics, rather than drug exposure, may account for the association [38].

Therefore, to date, despite the relevance of the topic, a comprehensive review investigating this topic is still needed.

To address this clinical and research question, we conducted a narrative review to collect the available literature investigating the relationship between AP use and bone fragility.

## 2. Materials and Methods

A review of the published literature was conducted and reported, screening the current available literature. PubMed/Medline, the Cochrane Database, and Scopus were searched in June 2024 with no lower date limit using the following search string with MeSH terms and keywords: (“antipsychotic” OR “neuroleptic”) AND (“risk fracture” OR “fracture risk” OR fracture OR “fragility fracture” OR “bone mass density” OR “bone mineral density” OR “BMD” OR “bone” OR “osteoporosis” OR “osteoblast” OR “osteoclast” OR “osteopenia” OR “bone loss” OR “bone health” OR “bone metabolism”). Two authors (GS and RdF) independently conducted all the searches and screened the titles and abstracts to identify articles for inclusion. Both reviewers reviewed the full text to reach a consensus on the inclusion or exclusion of the study when the decision could not be based on the title and abstract, contacting a third senior author (MM) in cases of major discrepancies. An additional screening of the references list of each included article and of the available gray literature at our institution was performed for the inclusion of potential additional articles.

### 2.1. Study Population and Study Design

Studies using any AP treatment for any psychiatric condition diagnosed according to the DSM-IV, DSM IV-TR, DSM-5 [39], DSM-5-TR [40], or ICD-10 or -11 criteria, or validated scales with cut-offs, or clinical records where the bone metabolism, osteoporosis, and/or risk of fractures had been assessed or evaluated were included. The patients of the included studies could be under chronic AP treatment or be diagnosed with an early phase of disease and could be drug-naive. Studies reporting on patients who exhibited general medical, neurological comorbidity or unclear or unverified diagnoses according to the ICD criteria were excluded. Studies involving a comparator group without exposure to APs were also considered.

All experimental and observational studies [including case–controls studies, cohort studies, and randomized controlled trials (RCTs)] written in English were assessed during the title, abstract, and full-text screenings and included. Narrative and systematic reviews, meta-analysis, umbrella reviews, expert opinions, editorials, letters to the editor, and book chapters were only considered for their discussion section. We considered studies evaluating only the point of view of a patient or caregiver as non-eligible for the present review.

### 2.2. Quality Assessment

The modified version of the Newcastle–Ottawa scale for cross-sectional studies was used independently by the same authors who performed data extraction (GS e RdF) to assess the quality of the selected studies (Table 1). A third author (MM) was involved to resolve disagreements between reviewers.

Based on the total score, quality was classified as “low” (0–3), “moderate” (4–6), or “high” (7–9). Criterion numbers (in bold) represented the following: 1, representativeness of the exposed cohort; 2, selection of the nonexposed cohort; 3, ascertainment of exposure; 4, demonstration that the outcome of interest was not present at the start of the study; 5, comparability of cohorts on the basis of the design or analysis; 6, assessment of outcome; 7, follow-up was long enough for outcomes to occur; 8, adequacy of follow-up of cohorts. Based on the Modified Newcastle–Ottawa scale rules, each study was awarded a maximum of one or two points for each numbered item within categories [14].

### 2.3. Outcome Measures

The evaluation of the BMD, bone metabolism, and bone health, in general, associated with concomitant AP use was the primary outcome. The fracture risk related to the use of any AP for any psychiatric condition was the secondary outcome. The evaluation of any specific demographic or clinical feature related to the increased fracture risk was also assessed.

## 3. Results

A total of 1707 relevant articles were identified through the initial search, 621 abstracts were screened, 86 full-text articles were assessed for eligibility based on our inclusion criteria, and 15 articles were excluded in the full-text phase [47,53,54,55,56,57,58,59,60,61,62,63,64,65,66], resulting in 15 studies that were eligible for the review (Table 2).

The included studies were published from 1997 to 2023; three were randomized-controlled trials, six were cross-sectional studies, and six were cohort studies, of which three were prospective and three were retrospective. Characteristics of included studies and their participants are summarized in Table 3, Table 4 and Table 5.

Three studies were conducted in Europe, nine in Asia, one in Turkey, one in Canada, one in Australia, and one in Saudi Arabia. A total of 3245 initial patients were identified, of whom 1153 patients were lost at the follow-up; 1357 patients underwent AP treatment, affected by schizophrenia.

The mean follow-up was 18.7 ± 9.4 months, ranging from a minimum of 4 months to a maximum of 60 months. There were 550 (40.4%) and 693 (59.6%) female patients in the treated and in the control group, respectively. The mean age of the treated patients was 43.8 ± 13.1 years, and 53.2 ± 15.3 years for the control group. The mean BMI was reported in fourteen studies, and it was 26.1 ± 3 and 23.9 ± 3.3 for the treated and control groups, respectively.

The mean AP treatment duration of the treated group was 15.8 ± 13.9 years. Six studies reported the BMD, expressed in g/cm^2^, for both case and control groups with various differences in values [35,42,46,50,52,67]. The femoral neck, lumbar spine, total femur, trochanter, and intertrochanter areas were the areas evaluated. Among the included studies, two reported a statistically significant difference in lumbar BMD reduction between the AP-exposed group and the control group [35,46]. On the other hand, three papers found a clear tendency but not statistical significance for lumbar BMD levels to decrease in the group of patients exposed to APs [42,52,67]. Femoral neck BMD levels had been reported in four [35,46,50,67] of the case–control studies [35,42,46,50,52,67]; two reported a statistically significant difference in femoral neck BMD reduction between the AP-exposed group and the control group [35,46]. Finally, six studies did not report a level of significance between groups [35,42,48,50,52,67]. Regarding bone health, 10 studies reported bone density data through BMD [22,35,42,44,46,47,50,51,52,67], while 6 reported this as a T-score [22,35,41,47,48,50] and 6 as a Z-score [22,43,45,48,49,51].

## 4. Discussion

Considering the widespread use of APs globally, together with their potential side effects related to reducing BMD and the consequential increased risk of bone fractures, the causality of this relationship has gained interest in the literature. Therefore, we run this narrative review with the aim to investigate the potential role of AP treatment in increasing the risk of osteoporosis. In this study, 15 articles were included in the qualitative analysis of fracture risk related to antipsychotics use. The included papers demonstrated a general tendency towards a reduction in the BMD in the studied areas, in the groups of patients exposed to APs, especially in the femoral neck area. These findings are of clinical relevance and suggest that BMD assessment could be useful for monitoring bone health in patients using APs who could also be referred early for orthopedic consultation as a possible prevention strategy.

AP medications have always been the subject of in-depth evaluations both from a clinical and research point of view, both with respect to their efficacy on psychotic symptoms and their safety and tolerability profile [68] even in orthopedic patients [69,70]. First-generation antipsychotics (FGAs), for example, have been studied at length for their cardiotoxic effects and for the risk of movement disorders, while second-generation antipsychotics (SGAs) have been extensively evaluated for the associated risk of metabolic syndrome, and third-generation antipsychotics have been for akathisia [68,71,72]. From this perspective, the effect on bone health, although it is well-known, has been neglected and in any case underestimated in the choice of antipsychotic therapy, passing into the background compared to other adverse events [63].

Osteoporosis is a skeletal disease leading to a weakness condition with reduced bone strength and mineral density, thus increasing the risk for fragility fractures, and consequently pain and physical impairment [73]. Severe mental and physical diseases and prolonged antidepressant prescription can also facilitate the progression of symptoms and the worsening of quality of life [13,74]. Indeed, a strong relationship exists between bone health, general health status, and quality of life [75]. In this context, fractures of the hip and vertebrae are associated with reductions in BMD and the resulting pain, inability to walk, and the reduction in the quality of life carry a high risk of adverse health outcomes and mortality [76,77]. It should also be considered that the costs of osteoporosis, osteopenia, and the socio-economic burden of managing the resulting fractures are estimated to grow worldwide [78]. Several theories have attempted to explain the relationship between APs and BMD reduction, but the most reliable seems to be the one linked to the mechanism connected to prolactin. Indeed, women suffering from hyperprolactinemia caused by pituitary adenomas have been shown to experience a reduction in cortical bone density, as well as a reduction in trabecular bone density [79,80]. The osteopenic levels of BMD in these hyperprolactinemic women were more than one standard deviation below the mean BMD of control groups, correlating with a twofold increase in fracture risk [10]. Prolactin secretion is normally inhibited by dopamine released from the hypothalamus into the tuberoinfundibular system, which acts on D_2_ receptors in lactotroph cells of the anterior pituitary. Dopamine is the primary hypothalamic inhibitor of prolactin release. Conventional APs raise prolactin levels by blocking this dopaminergic inhibition. Even low doses of APs, such as haloperidol (0.5–1.5 mg), can cause a significant increase in prolactin levels within 1–2 h [81]. Indeed, concerns exist about BMD in patients with schizophrenia treated with prolactin-elevating medications, and this issue seems to be confirmed by our study. Besides the risk associated with APs, schizophrenia patients are at increased risk of osteoporosis due to factors such as limited exercise, poor nutrition, smoking, and excessive water intake. On the other hand, obesity may offer some protective effects [81].

In our study the majority of the patients who experienced reduced BMD were men, treated with APs, middle-aged adults, and treated for an average of 15.8 years. These results are in line with similar scattered data reported in the literature, where a study demonstrates that over 60% of patients taking psychiatric medications experience bone loss, causing osteopenia and in some cases osteoporosis, with osteopenia being more prevalent, and the majority of those with low bone mass were in their forties. Still, males usually outnumber females [41]. Downs et al. reported that 70% of patients on AP medications developed osteoporosis, with the risk influenced by the duration and dosage of the medication [82]. However, Kishimoto et al. found that all of their patients, across all age groups, had low BMD compared to healthy individuals, regardless of the dosage or duration of AP use [83]. In this study, age was identified as a factor potentially influencing the relationship between AP use and BMD. Indeed, women under 60 taking APs had lower BMD compared to non-users, putting them at a higher risk of developing osteoporosis [84]. Several factors may explain this association. Peak bone mass is typically reached by age 30, after which BMD begins to decline. Since psychiatric disorders, particularly schizophrenia, often emerge during adolescence or early adulthood—coinciding with the initiation of AP treatment and unhealthy lifestyle choices like smoking—it is possible that peak bone mass is compromised [85]. Additionally, it has been suggested that tolerance to the effects of APs may develop with age, or that younger women may be more sensitive to the prolactin-elevating effects of these medications [66].

A previous systematic review with meta-analysis provides low-quality evidence suggesting that APs may increase fracture risk, with an estimated 57% increase in hip fractures and a 17% increase in overall fractures [27]. The effect was consistent across various methodological factors (risk of bias, effect measures, study design), patient characteristics (underlying medical conditions, time to fracture after treatment initiation, sex), and treatment characteristics (dose, prolactin elevation, FGA or SGA, and specific agents). Therefore, authors conclude that they had sufficient data to compare the effects of four individual agents—haloperidol, risperidone, olanzapine, and quetiapine—but found no significant differences among them. For other agents, there was insufficient data for meaningful comparisons. Moreover, the authors rated the quality of evidence using the GRADE approach and concluded that the overall evidence was of low quality, proposing caution in interpreting the results as between-study heterogeneity limited the confidence in their estimate.

Another point of discussion opened by our work is the risk/benefit ratio of the long-term use of APs. In fact, if the benefit of off-label use or use as an the add-on therapy of bipolar disorder and major depressive disorder does not appear to be so clear, the issue related to schizophrenia appears to be more complex, also because of the consequential risk of osteoporosis, which in any case increases with the chronological age of the patients [86,87]. In this regard, clinical guidelines lack systematic recommendations for whether to continue or discontinue treatment beyond 1–2 years, though they highlight the risks of relapse associated with stopping treatment [88,89]. Therefore, the long-term effects of AP treatments, including reduced BMD, beyond the first two years are not well understood, largely due to the absence of double-blind, placebo-controlled randomized trials. An emerging body of the literature has begun to question the necessity of long-term AP use. Studies involving long-term AP exposure in animals, naturalistic cohorts, and treatment discontinuation have been cited by some researchers arguing that APs do not necessarily improve outcomes in the long term and may even have iatrogenic adverse effects [90,91]. Therefore, it is not yet clear whether the use of antipsychotic drugs can have a direct influence on bone density, even in the absence of risk conditions, just as it is not clear whether chronic use determines their dangerousness and, if so, after how long the risk increases significantly, such that the disadvantages could outweigh the efficacy data.

However, others argue that evidence for iatrogenic effects remains insufficient [92]. This ongoing debate, combined with the inherent biases in interpreting long-term studies, leaves clinicians with unclear guidance on the matter [93]. These data are particularly relevant when we consider that we found a mean AP treatment duration of the treated group of 15.8 ± 13.9 years. Despite the indications of short-term studies and guidelines, the data collected from the literature demonstrate how in the real world it is then essential to use, even constantly, antipsychotics for decades [94]. Osteoporosis, together with all other potential adverse events, must therefore be included and considered by authors and clinicians, but its relative risk must not be a prejudicial factor on the use of APs in schizophrenia.

It is also interesting to analyze the geographical data regarding the use of APs. In fact, if on the one hand APs have had a widespread and global diffusion for many years now, the availability of new second- and third-generation molecules and long-acting formulations is not so widespread, mainly for pharmacoeconomic reasons [95]. According to a recent study evaluating the international trends in APs’ use through a repeated cross-sectional design, analyzing data from 16 countries between 2005 and 2014 [2], over the study period, the overall prevalence of antipsychotic use increased in 10 out of the 16 countries. In 2014, Taiwan had the highest prevalence of antipsychotic use (78.2 per 1000 individuals), while Colombia had the lowest (3.2 per 1000). Among children and adolescents (0–19 years), antipsychotic use varied from 0.5 per 1000 in Lithuania to 30.8 per 1000 in Taiwan. For adults (20–64 years), the rates ranged from 2.8 per 1000 in Colombia to 78.9 per 1000 in the publicly insured population in the United States. Among older adults (65+ years), use ranged from 19.0 per 1000 in Colombia to 149.0 per 1000 in Taiwan. The use of SGAs increased across all populations, with the atypical-to-typical ratio ranging from 0.7 in Taiwan to 6.1 in New Zealand and Australia. The most frequently prescribed APs were quetiapine, risperidone, and olanzapine. There were significant variations in the prevalence and patterns of APs’ use between countries. In most populations, AP use, particularly that of SGAs, increased over time. The authors conclude that the high rates of AP prescriptions they found among older adults and youths in some countries warrant further investigation and systematic pharmacoepidemiologic monitoring [2]. These data are in line with our results, considering that we included three studies conducted in Europe, nine in Asia, one in Turkey, one in Canada, one in Australia, and one in Saudi Arabia. This can be explained by the greater scientific productivity in Europe and Asia, but this would not explain the absence of the USA among the included articles [96], while it would be much more understandable to integrate the data of the greater use of FGAs in Asian countries compared to the rest of the world, with an increased risk of inducing iatrogenic osteoporosis or worsening the pre-existing one [2].

In this context, the therapy currently evaluated is based on the mainstream dopaminergic hypothesis of schizophrenia, which is currently treated with D_2_-blocking antipsychotic drugs, as explored in our review [97]. However, if this association were confirmed, this risk of reduced BMD might not be so marked in the perspective of new lines of research. In fact, antipsychotic drugs exploiting the glutamatergic or muscarinic hypothesis of schizophrenia are currently under development, bypassing, at least conceptually, a classical D_2_ receptor blockade and greatly reducing the risk of hyperprolactinemia as well as iatrogenic osteoporosis [98,99,100].

However, the link between APs and bone fragility is still highly debated. Indeed, in their meta-analysis of 19 observational studies, Lee and colleagues showed that the use of any AP was linked to a nearly 1.5-fold increase in fracture risk, regardless of study design, fracture site, or age group [101]. The risk was higher among elderly individuals, for hip fractures, and in populations with a high baseline fracture incidence. While previous studies have disagreed on which types of AP medications are linked to fracture risk, their analysis confirmed that both FGAs and SGAs are associated with this risk. Still, among individual agents, chlorpromazine appeared to carry the highest risk, while risperidone was linked to the lowest risk. These findings, similarly to our results, are also consistent with two previous meta-analyses that demonstrated a significant association between AP use and increased fracture risk. Takkouche et al. reviewed 12 studies and reported a pooled odds ratio (OR) of 1.59 (95% CI 1.27–1.98) for patients using either FGAs or SGAs [102]. A subsequent review by Oderda differentiated the risk of fracture associated with FGAs (OR 1.68, 95% CI 1.43–1.99) and SGAs (OR 1.30, 95% CI 1.14–1.49) [103]. Therefore, our updated review builds on these findings by analyzing 15 eligible studies and identifying key sources of heterogeneity through thorough qualitative investigation.

Finally, a less discussed hypothesis that deserves to be mentioned is also linked to the idea that it is not the antipsychotics themselves that increase the risk of osteoporosis and fractures, but rather the severity of the psychiatric illness that impacts the quality of life of affected patients, reducing physical activity, quality of nutrition, adherence to drug therapy, and general life expectancy [104]. In this regard, patients with severe mental illnesses tend to experience a lower quality of life compared to the general population. Their conditions can hinder their ability to complete daily tasks, leading to reduced independence and diminished activities to improve their general health status, such as taking medications, having frequent check-ups, reducing alcohol and fatty food intake, exercising frequently, and quitting smoking. Globally, when conducting studies on the impact of drug therapies, the impact on the control of risk factors associated with those same pathologies should also be considered [22,105].

Last but not least, the complexity of polypharmacy must always be considered, including the prescription of benzodiazepines and drugs potentially impacting bone health, such as oral corticosteroid use or other high-risk medications (e.g., thyroid hormone replacement, immunosuppressant drugs, warfarin) [15].

A number of limitations should be taken into account when interpreting the results of our review. Firstly, our analysis included studies examining all potential APs, without limitations, both in monotherapy and in combination, but we did not find any studies involving newer APs like lurasidone, cariprazine, and brexpiprazole, despite their inclusion in our search criteria. Moreover, some studies were found to be poorly informative with respect to the type of AP used, or presented only aggregated category data, reducing the final quality.

Second, although a large number of articles were initially identified, the screening process resulted in only a small subset being selected for qualitative and quantitative evaluation. This issue can be attributed to the high variability and heterogeneity of the collected data, which hindered further generalization and data aggregation. Furthermore, the heterogeneity of the studies included in the quantitative analysis, combined with insufficient identification of overlapping characteristics among the studied populations, prevented specific analyses of demographic factors’ (e.g., gender, age) or clinical factors’ (e.g., age of onset, disease duration) influences on fracture risk. These remain important areas for future exploration. Still, it should be noted that when analyzing time- and aging-related phenomena such as the development of osteopenia and osteoporosis, on the one hand, there is the direct effect of the use of implicated drugs such as antipsychotics in the long term, and on the other hand, the aging of patients itself increases the risk of reduced BMD, therefore both the use of comparison populations followed over time and the correction for age should be performed.

Lastly, the lack of detailed data and the methodological differences among the included studies limit our ability to draw definitive conclusions, instead highlighting a risk trend without pinpointing a clear biological mechanism. Nevertheless, an unrestricted literature review concerning the target population (e.g., psychiatric disease), intervention (e.g., AP characteristics), comparator, or outcome was conducted, aiming for results that are as naturalistic as possible while excluding only significant general medical comorbidities that might affect fracture outcomes.

## 5. Conclusions

According to the results collected in this review, prolonged use of AP treatment seems to be associated with an increased risk of reduced BMD, and, consequentially, with an augmented risk of bone fragility and fractures. In the literature, it is known that this effect is particularly marked in vulnerable groups, such as those with significant medical comorbidities, the elderly, and postmenopausal women, but our results demonstrated that it may also apply to anyone using APs in the long-term, regardless of starting risk level.

Clinical pharmacovigilance data relating to the bone effects of APs are not intended to discourage their use, especially in conditions of psychosis or resistance to first-line treatment, but rather to inform clinicians in order to make informed and conscious decisions, minimizing the risks and enhancing their effectiveness. In this light, routine BMD assessment could be useful to monitor bone health in patients using APs who could also be referred for orthopedic consultation as a possible prevention strategy.

## Figures and Tables

**Table 1:** Quality assessment of included studies according to the Modified Newcastle–Ottawa scale.

	Criteria								Total	Quality
	1	2	3	4	5	6	7	8		
Al-Omran et al. (2016) [41]	2	1	1	1	1	1	1	1	9	High
Bilici M et al. (2002) [42]	1	2	1	1	1	1	1	1	9	High
Bulut SD et al. (2016) [35]	1	2	1	1	1	1	1	1	9	High
Howes OD et al. (2005) [43]	1	0	1	1	1	1	1	1	7	High
Keely EJ et al. (1997) [44]	1	0	1	1	1	1	1	1	7	High
Lee TY et al. (2010) [45]	0	0	1	1	1	2	1	1	7	High
Liang M et al. (2019) [46]	1	1	1	1	1	2	1	1	9	High
Liu F et al. (2023) [47]	1	0	1	1	1	1	1	1	7	High
Lin CH et al. (2019) [48]	1	1	1	1	1	1	1	1	8	High
Manavi BA et al. (2023) [7]	1	1	1	1	1	2	1	1	9	High
Meaney AM et al. (2004) [49]	1	0	1	1	1	1	1	1	7	High
Qiu J et al. (2020) [50]	1	1	1	1	1	1	1	1	8	High
Takahashi T et al. (2013) [51]	2	1	1	1	1	1	1	1	9	High
Yang J et al. (2011) [22]	1	0	1	1	1	1	1	1	7	High
Wang M et al. (2014) [52]	2	1	1	1	1	1	1	0	8	High

**Table 2:** Included studies.

Authors	Journal	Publication Year	Type of Study
Al-Omran et al. [41]	Saudi J Med Med Sci	2016	prospective
Bilici M et al. [42]	Int J Neurosci	2002	retrospective
Bulut SD et al. [35]	Psychiatr Danub	2016	retrospective
Howes OD et al. [43]	J Clin Psychopharmacol	2005	prospective
Keely EJ et al. [44]	Endocr Pract	1997	cross-sectional
Lee TY et al. [45]	Psychiatry Investig	2010	cross-sectional
Liang M et al. [46]	Int J Endocrinol	2019	cross-sectional
Liu F et al. [47]	BMC Psychiatry	2023	cross-sectional
Lin CH et al. [48]	Sci Rep	2019	cross-sectional
Manavi BA et al. [7]	Front Psychiatry	2023	cross-sectional
Meaney AM et al. [49]	Br J Psychiatry	2004	retrospective
Qiu J et al. [50]	Arch Osteoporos	2020	retrospective
Takahashi T et al. [51]	Schizophr Res	2013	prospective observational
Yang J et al. [22]	Psychiatry Investig	2011	prospective controlled
Wang M et al. [52]	Hum Psychopharmacol	2014	prospective

**Table 3:** Demographic characteristics of included studies.

Authors	Treated Patients *n*	Controls *n*	Age of Treated Patients (Mean)	SD	Age of Patients Control Group (Mean)	SD
Al-Omran et al. [41]	145	150	40.8	7.2	45.3	6.5
Bilici M et al. [42]	75	20	29.6	6.5	31.1	7.7
Bulut SD et al. [35]	42	39	37.9	10.4	35.8	10.1
Howes OD et al. [43]	102	NA	46	13.1	NA	NA
Keely EJ et al. [44]	16	NA	41.3	2.9	41	3.1
Lee TY et al. [45]	45	NA	49.5	11.1	NA	NA
Liang M et al. [46]	101	71	26.1	4.9	25.9	4.7
Liu F et al. [47]	211	NA	48.6	12.3	NA	NA
Lin CH et al. [48]	111	44	41.1	8.1	40.2	8.6
Manavi BA et al. [7]	49	571	52.6	6.8	64.3	5.3
Meaney AM et al. [49]	55	NA	50.5	12	NA	NA
Qiu J et al. [50]	82	43	57	2.8	NA	NA
Takahashi T et al. [51]	141	23	58.3	10.7	59.9	12.6
Yang J et al. [22]	19	NA	39.68	10.2	NA	NA
Wang M et al. [52]	163	90	34.5	10.4	34.2	10.6
Total	1357	1051	43.8	13.1	53.2	15.3

Note: NA: not available.

**Table 4:** Psychiatric condition and antipsychotic drug analyzed in the included studies.

Authors	Psychiatric Condition	Antipsychotic Drug
Al-Omran et al. [41]	psychiatric illness	antipsychotic medications
Bilici M et al. [42]	schizophrenia	classical vs. atypical
Bulut SD et al. [35]	schizophrenia	olanzapine—risperidone—quetiapine—haloperidol
Howes OD et al. [43]	psychotic disorder	haloperidol—risperidone—quetiapine—olanzapine—pimozide—pipotiazine—sulpiride
Keely EJ et al. [44]	schizophrenia	trifluoperazine—fluphenazine enanthate— fluphenazine—decanoate—fluphenthixol—haloperidol—chlorpromazine
Lee TY et al. [45]	schizophrenia	risperidone—olanzapina—clozapine
Liang M et al. [46]	schizophrenia	olanzapine
Liu F et al. [47]	schizophrenia	fluphenazine—risperidone—haloperidol— amisulpiride—sulpiride—paliperidone—clozapine—mirtazapine—olanzapine—quetiapine— aripiprazole
Lin CH et al. [48]	schizophrenia	clozapine
Manavi BA et al. [7]	schizophrenia	quetiapine—aripiprazolo—clozapine—paliperidone—risperidone—trifluoperazine
Meaney AM et al. [49]	schizophrenia	chlorpromazine—haloperidol—risperidone— olanzapine—flupentixol—pluphenazine—sulpiride
Qiu J et al. [50]	schizophrenia	risperidone—amisulpride—paliperidone— first-generation antipsychotics
Takahashi T et al. [51]	schizophrenia	first-generation antipsychotic—risperidone— blonanserine—aripiprazole—olanzapine— perospirone—quetiapine
Yang J et al. [22]	bipolar disorder	risperidone—olanzapine—quetiapine
Wang M et al. [52]	schizophrenia	perphenazine—sulpiride—chlorpromazine— clozapine—quetiapine—aripiprazole

**Table 5:** Doses and treatment duration of antipsychotic drugs.

First Author	Antipsychotic	Mean Dose (mg/Day)	SD	Subjects (N)	Mean Duration Treatment (Years)	SD
Al-Omran [41]	total	NS	NS	145	8.4	6.4
Bilici M [42]	total	63.8	NS	75	13.5	3
	haloperidol	15	NS	20	13.5	3
	flufenazin	4	NS	10	13.5	3
	zuclopentixol	50	NS	10	13.5	3
	olanzapin	10	NS	10	13.5	3
	risperidon	4	NS	20	13.5	3
	clozapin	300	NS	5	13.5	3
Bulut [35]	total	489.6	291.1	42	60.1	68.7
	PSG (haloperidol risperidone)	532.4	373.9	19	24.6	16.9
	PRG (olanzapine— quetiapine)	454.3	201.6	23	95.6	120.6
Howes OD [43]	total	356	318	102	3	8.5
Keely EJ [44]	total	NS	NS	16	18	7.3
Lee TY [45]	total	280.1	71.0	45	24.7	9.3
	risperidone	252.5	74.8	20	23.8	8.8
	olanzapine	273.7	83.7	15	27.3	11.3
	clozapine	265.0	35.7	10	22.6	6.9
Liang M [46]	olanzapine	452.8	129.2	101	2.4	1.1
Liu F [47]	total	700	400	211	21.8	11.4
Lin CH [48]	non-clozapine	586.6	454.5	69	7.8	6.9
	clozapine	420.5	173.8	42	9.4	3.6
Manavi [7]	total	NS	NS	NS	37.4	46.4
Meaney AM [49]	total	500.1	474.6	55	18.3	7.7
Qiu J [50]	PRG	469.2	261.3	43	27.4	12.5
	clozapine + PRG	397.3	233.1	82	27.4	12.5
Takahashi T [51]	total	497.6	387	164	26.7	13.8
	PRG	519.4	412.0	141	27.4	13.7
	PSG	363.7	164.4	23	22.1	13.8
Yang J [22]	risperidone	0.5–1.5	0.2	9	49.5	23.5
	olanzapine	2.5–5	0.6	4	49.5	23.5
	quetiapine	25–100	18.8	6	49.5	23.5
Wang M [52]	chlorpromazine	325.0	47.6	14	12.9	3.1
	perphenazine	30	7.8	13	12.9	3.1
	sulpiride	800	144	12	12.9	3.1
	clozapine	318.7	21.1	14	12.1	2.3
	quetiapine	650	62.4	12	12.1	2.3
	aripiprazole	18.2	7.8	12	12.1	5

PRG: prolactin-raising group, PSG: prolactin-sparing group, NS: nonspecific.

## Data Availability

The data presented in this study are available on reasonable request from the corresponding author.
